# Manifestations of left ventricular dysfunction and arrhythmia in patients with chronic hypoparathyroidism and pseudohypoparathyroidism: a preliminary study

**DOI:** 10.1186/s12902-020-0541-6

**Published:** 2020-05-11

**Authors:** Yabing Wang, Kun He, Ou Wang, Xue Lin, Sixing Chen, Yan Jiang, Mei Li, Weibo Xia, Xiaoping Xing

**Affiliations:** 1 Department of Endocrinology, Key Laboratory of Endocrinology of the Ministry of Health, Peking Union Medical College Hospital, Chinese Academy of Medical Science & Peking Union Medical College, Dongcheng District, Shuaifuyuan No.1, Beijing, 100730 China; 2 Department of Internal Medicine, Peking Union Medical College Hospital, Chinese Academy of Medical Sciences & Peking Union Medical College, Dongcheng District, Shuaifuyuan No.1, Beijing, 100730 China; 3 Department of Cardiology, Peking Union Medical College Hospital, Chinese Academy of Medical Sciences & Peking Union Medical College, Dongcheng District, Shuaifuyuan No.1, Beijing, 100730 China

**Keywords:** Hypoparathyroidism, Pseudohypoparathyroidism, Heart, Function, Arrhythmia

## Abstract

**Background:**

Cardiac damage triggered by severe hypocalcemia is well known. However, the role of chronic hypoparathyroidism (HP) and pseudohypoparathyroidism (PHP) in cardiac health is still unclear. We investigated the effect of chronic HP and PHP on cardiac structure and conductive function in patients compiling with treatment.

**Methods:**

The study included 18 patients with HP and eight with PHP aged 45.4 ± 15.4 and 22.1 ± 6.4 years, respectively with a previously regular follow-up. In addition, 26 age- and sex-matched healthy controls were included. General characteristics and biochemical indices were recorded. Cardiac function and structure were assessed by estimation of myocardial enzymes, B-type natriuretic peptide (BNP), and echocardiography. The 12-lead electrocardiogram and 24-h Holter electrocardiography were performed to evaluate the conductive function.

**Results:**

Levels of serum calcium in HP and PHP were 2.05 ± 0.16 mmol/L and 2.25 ± 0.19 mmol/L, respectively. The levels of myocardial enzyme and BNP were within the normal range. Adjusting for age at evaluation and body mass index, all M-mode measurements, left ventricular mass (LVM), LVM index (LVMI) and relative wall thickness (RWT) were comparable between patients and controls. Prolongation of corrected QT (QTc) intervals occurred in 52.6% (10/19) of patients, and 6.7% (1/15) of patients manifested more than 100 episodes of supraventricular and ventricular extrasystoles, as well as supraventricular tachycardia. None of the above arrhythmias was related to a severe clinical event.

**Conclusions:**

From this pilot study, patients diagnosed with HP and PHP and well-controlled serum calcium levels manifested normal cardiac morphology and ventricular function, except for prolonged QTc intervals, and a small percentage of mild arrhythmias needing further investigation.

## Background

Hypoparathyroidism (HP) and pseudohypoparathyroidism (PHP) are rare diseases due to parathyroid hormone (PTH) insufficiency or resistance leading to hypocalcemia, hyperphosphatemia and related symptoms. The conventional treatment for HP and PHP includes calcium supplementation combined with vitamin D analogs. To avoid hypercalciuria-related renal damage and maintain optimal levels of calcium phosphate synthesis, the serum calcium levels were regulated slightly below normal or within the low-normal range in HP patients. Hyperphosphatemia often persists among HP and PHP patients receiving conventional therapy.

High serum phosphorus directly triggers vascular injury, increases the mineral deposition in vascular smooth muscle cells in studies in vitro [1], and increases vascular calcification in individuals with or without chronic kidney disease [2], which indicates a high risk of cardiovascular diseases (CVDs) [3]. Serum calcium plays an important role in excitation-contraction coupling of the myocardium, and the influx of extracellular calcium largely initiates and determines the degree of myocardial contraction. Chronic hypocalcemia and hyperphosphatemia have been reported as risk factors for CVDs among different populations [4, 5]. Cardiac abnormalities, such as acute heart failure, dilated cardiomyopathy, Takotsubo cardiomyopathy and arrhythmia with abnormal cardiac structure, have been reported in patients diagnosed with HP and PHP combined with chronic and severe hypocalcemia due to irregular treatment [6–8]. However, few studies have reported cardiovascular morbidities in patients with chronic HP and PHP and relatively stable levels of serum calcium [9–12]. Meanwhile, the long-term influence of chronic HP or PHP on cardiac morphology, function and conduction is still unclear, especially among patients receiving regular treatment.

Thus, the objective of the current pilot study was to directly investigate the effect of long-standing HP and PHP on cardiac morphology and function, and cardiac conductive system in patients compling with treatment of calcium and vitamin D analogs.

## Methods

### Subjects and controls

Between October 2017 and April 2018, among nearly 120 patients diagnosed with HP and PHP with a regular follow-up at the Metabolic Bone Disease Outpatient Clinic, Department of Endocrinology of Peking Union Medical College Hospital (PUMCH) in Beijing, a total of 28 subjects who agreed to participate in this study were continuously subjected to echocardiography. Patients with cardiac arrhythmia before the diagnosis of hypoparathyroidism (*n* = 1) and those diagnosed with post-surgical hypoparathyroidism due to parathyroidectomy (*n* = 1) were excluded. The remaining 26 patients and 26 age- and sex-matched healthy controls with normal thyroid function and with no CVD or associated risk factors during the same period were finally included in the analysis. The controls came from the health examination center in PUMCH. The patients were evaluated with a 12-lead electrocardiogram (ECG), cardiac markers, echocardiography and 24-h Holter electrocardiography. The controls were evaluated with echocardiography by the same physician as the patient group. All patients received calcium combined with vitamin D analogs (plain vitamin D or calcitriol) at the time of investigation independently to enrollment. The study was conducted according to the principles of the Declaration of Helsinki and approved by the Ethics Committee of PUMCH. Written informed consent was obtained from all included subjects or the parents for subjects aged below 18 years. The diagnosis of HP and PHP was established by the presence of hypocalcemia, and hyperphosphatemia with inappropriately low and normal-to-high intact PTH levels, respectively, while the secondary hyperparathyroidism including chronic kidney disease and vitamin D deficiency (serum 25OHD < 20 ng/ml) [13] was excluded for PHP. All the patients had normal kidney function evaluated by estimated glomerular filtration rate (eGFR) and no history of chronic malabsorption or diarrhea.

### Clinical parameters

Clinical demographics including diagnosis, gender, age of symptom onset, course, age of cardiac evaluation, and comorbidities (including hypertension, diabetes mellitus, and coronary artery disease (CAD)) were self-reported and confirmed by medical records. Parameters such as height, weight, and right arm systolic and diastolic blood pressure were measured by a single experienced physician. Body mass index (BMI) was calculated as weight in kilograms divided by height in squared meters. Body surface area (BSA) was calculated using the formula of Du Bois D and Du Bois EF [14]. Symptoms of dyspnea, shortness of breath, palpitations, history of syncope, chest pain, and edema of extremities were self-reported by patients, and evaluated and established by the same experienced physician.

### Laboratory parameters

Venous blood samples were obtained in the morning after an overnight (≥ 10 h) fasting and analyzed during echocardiography and electrocardiography. The concentrations of total serum calcium (Ca), serum phosphorus (P), potassium, magnesium, serum 25(OH) D, glucose, triglycerides, total cholesterol, low-density lipoprotein cholesterol, high-density lipoprotein cholesterol, and creatinine were measured using an automated biochemical analyzer (Beckman Coulter AU5800, USA). Serum intact PTH was measured based on a chemiluminescence immunoassay (Siemens ADVIA Centaur, Germany). Serum P was expressed as multiples of age-specific upper limit of normal range (×ULN). The eGFR was calculated using the abbreviated Modification of Diet in Renal Disease Study formula [15]. Creatine kinase (CK), creatine kinase-myocardial isoenzyme (CKMB), cardiac troponin I (cTnI), and B-type natriuretic peptide (BNP) were measured simultaneously. The intra-assay coefficients of variations (CVs) in 25(OH) D and PTH were 5.9 and 2.6%, with inter-assay CVs 6.5 and 5.8%, respectively. The intra-assay and inter-assay CVs of other biochemical parameters were all < 3.5%. The controls self-reported that their levels of serum calcium were normal on their previous routine physical examination, which were not re-examined at the time of echocardiography examination.

### Echocardiography

All the included subjects were evaluated using a non-invasive transthoracic echocardiogram (VIVID E9, GE Ultrasound, USA) with an m5s-D probe. M-mode measurements and conventional Doppler echocardiographic examinations were performed according to the American Society of Echocardiography (ASE) guidelines [16]. The parameters of interventricular septum end-diastolic thickness (IVSd), left ventricular posterior wall end-diastolic thickness (LVPWd), left ventricular end-diastolic diameter (LVEDD), and left ventricular ejection fraction (LVEF) were measured. Early diastolic mitral inflow velocity (E) and late diastolic mitral inflow velocity (A) were assessed in the Doppler-mode. Left ventricular mass (LVM, grams) was calculated using the ASE method [16]: 0.8 × {1.04 [(IVSd + LVEDD + LVPWd)^3^ – (LVEDD)^3^]} + 0.6. The LVM index (LVMI, g/m^2^) was calculated by dividing LVM by the BSA. The relative wall thickness (RWT) was calculated using the formula: (2 × LVPWd) / LVEDD.

### Electrocardiogram

Resting standard 12-lead ECG was obtained only in 19 patients. Resting heart rate, PR intervals, QRS duration, and corrected QT (QTc) intervals were determined via ECG analysis.

### 24-h Holter electrocardiography

Baseline cardiac rhythm and potential arrhythmias were evaluated using an ambulatory 12-lead electrocardiography in 15 patients over a 24-h period during their normal daily activities (MIC-Dream, JincoMed, Beijing, China). The total 24-h ventricular rates, and the maximal and minimal ventricular rates were measured automatically.

### Statistical analyses

Data were analyzed using SPSS software version 22.0 (Chicago, IL, USA). Categorical variables are expressed as numbers or percentages. Continuous variables are expressed as mean ± standard deviation or median and inter-quartile ranges, as appropriate. The clinical demographics and laboratory results of patients were compared with those of the healthy controls, and between patients diagnosed with HP and PHP using the Student’s t-test or Wilcoxon test. The cardiac parameters were compared using a general linear model adjusted for age at the evaluation, gender and other confounders. Associations between serum calcium, serum phosphorus, PTH and cardiac parameters were evaluated via partial correlation analysis.

## Results

### Demographic and laboratory characteristics of the 26 patients

The study included 18 patients diagnosed with HP and eight patients with PHP. The patients with HP included three patients with secondary HP following thyroidectomy, which was defined as post-surgical HP. The average age of onset of hypocalcemic symptoms and age at the cardiac evaluation were higher in HP subjects compared with PHP subjects (age of onset 23.9 ± 13.8 vs. 12.9 ± 7.4 years, *P* = 0.045; age at cardiac evaluation 45.4 ± 15.4 vs. 22.1 ± 6.4 years, *p* <  0.001, respectively). The disease courses from onset of hypocalcemia were longer in patients diagnosed with HP compared with those with PHP at the evaluation. One patient with HP suffered from diabetes, and four patients with HP had hypertension. No subject had self-reported CAD or related symptoms. The average levels of serum calcium were 2.05 ± 0.16 mmol/L in HP patients and 2.25 ± 0.19 mmol/L in patients with PHP (*P* = 0.011) during the evaluation. Serum magnesium, serum potassium, fasting blood glucose, and lipid components were within normal limits. The serum phosphorus levels (×ULN) were higher (1.13 ± 0.10 and 0.99 ± 0.12, *P* = 0.003) and the eGFR was lower (90.4 ± 21.1 and 127.3 ± 29.7 mL/min/1.73m^2^, *P* = 0.005) in HP than in PHP (Table 1).

**Table 1:** General characteristics of HP and PHP subjects

Characteristic	HP *N* = 18	PHP *N* = 8	*P*
Female, n (%)	12 (66.7%)	7 (87.5%)	0.375
Age of onset (y)	23.9 ± 13.8	12.9 ± 7.4	0.045
Course (y)	22.5 ± 15.8	9.2 ± 11.9	0.046
Age of heart evaluation (y)	45.4 ± 15.4	22.1 ± 6.4	0.000
BMI (kg/m^2^)	25.1 ± 3.9	22.2 ± 2.6	0.074
Diabetes, n	1	0	0.692
CAD, n	0	0	NA
Hypertension, n	4	0	0.205
Systolic BP (mmHg)	119.4 ± 12.0	107.6 ± 13.8	0.040
Diastolic BP (mmHg)	74.4 ± 9.0	72.9 ± 11.1	0.714
Serum calcium (mmol/L)	2.05 ± 0.16	2.25 ± 0.19	0.011
Serum phosphorus (×ULN)	1.13 ± 0.10	0.99 ± 0.12	0.003
Serum magnesium (mmol/L)	0.82 ± 0.07	0.85 ± 0.08	0.464
Serum potassium (mmol/L)	4.07 ± 0.26	4.10 ± 0.31	0.770
25(OH)_2_D (ng/mL)	23.8 [17.7, 41.4]	32.0 [19.9, 83.4]	0.500
Fasting blood glucose (mmol/L)	5.3 ± 0.4	4.9 ± 0.4	0.020
PTH (pg/mL)	3 [2.6, 7.9]	237.4 [43.9482.9]	0.000
ALP (×ULN)	0.54 ± 0.17	0.71 ± 0.37	0.111
β-CTX (×ULN)	0.74 ± 0.32	1.37 ± 0.69	0.008
eGFR (MDRD) (mL/min/1.73m^2^)	90.4 ± 21.1	127.3 ± 29.7	0.005
Total cholesterol (mmol/L)	4.6 ± 0.7	3.7 ± 2.0	0.178
Triglycerides (mmol/L)	1.3 ± 0.9	0.7 ± 0.6	0.181
LDL-C (mmol/L)	2.7 ± 0.4	2.2 ± 1.3	0.361
HDL-C (mmol/L)	1.1 ± 0.2	1.0 ± 0.5	0.619
CK (U/L)	126.5 [63.8, 145.8]	85.0 [67.5, 118.3]	0.153
CKMB (μg/L)	0.40 [0, 0.80]	0.45 [0.03, 0.65]	0.776
cTnI (μg/L)	< 0.017	< 0.017	–
BNP (ng/L)	13.0 [8.0, 25.8]	6.5 [3.3, 11.8]	0.070

Date represent the means (SD), median (P25, P75), or N (%)

Abbreviations: *HP* hypoparathyroidism; *PHP* pseudohypoparathyroidism; *BMI* body mass index; *CAD* coronary artery disease; *BP* blood pressure; *PTH* parathyroid hormone; *ALP* alkaline phosphatase; *β-CTX* C-terminal telopeptide of type I collagen; *eGFR* estimated glomerular filtration rate; *LDL-C* low-density lipoprotein cholesterol; *HDL-C* high-density lipoprotein cholesterol; *CK* creatine kinase; *CKMB* creatine kinase-myocardial isoenzyme; *cTnI* cardiac troponin I; *BNP* B-type natriuretic peptide

### Cardiac morphology and function in patients with HP and PHP

**Fig. 1:**
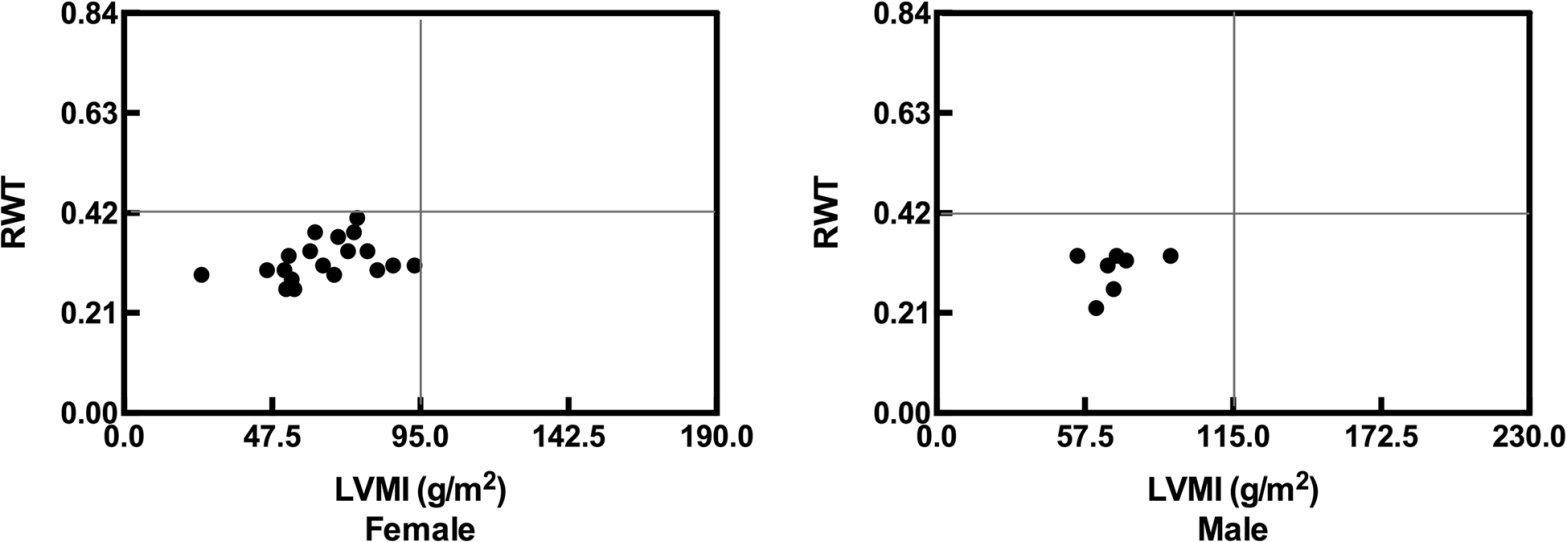
Cardiac geometry of female (*n* = 19) and male (*n* = 7) patients. Normal range of RWT was < 0.42 in both females and males, and the normal range of LVMI was < 95.0 g/m^2^ in females and < 115.0 g/m^2^ in males according to the American Society of Echocardiography guidelines. Abbreviations: *LVMI*: left ventricular mass index; *RWT*: relative wall thickness

The cardiac parameters of patients subjected to echocardiography were compared with age- and sex-matched healthy controls, as shown in Table 2. Adjusting for the age at evaluation and BMI, LVEF, and LVEDD, which reflect left ventricular systolic function, were comparable between patients and controls. The mean left ventricular ejection fractions were 68.4 ± 6.4% and 66.5 ± 7.4% in HP and PHP patients, respectively. Parameters reflecting left ventricular structure including IVSd, LVPWd, LVM, LVMI, and RWT were comparable between patients and controls. Among the 26 patients, LVMIs were less than 95 g/m^2^ in females, and less than 115 g/m^2^ in males. The RWT values were less than 0.42. According to the ASE guidelines, all patients displayed normal cardiac geometry (Fig. 1), except for one female patient with non-surgical HP with an RWT of 0.41 and another female with post-surgical HP with LVMI 93.2 g/m^2^ indicating potentially borderline concentric remodeling and eccentric hypertrophy, respectively. Levels of CK, CKMB, cTnI and BNP were within the normal ranges in both patients with HP and PHP.

**Table 2:** Comparison of echocardiographic parameters across different subgroups

Variables	HP *N* = 18	Control A *N* = 18	PHP *N* = 8	Control B *N* = 8	P^a^ value	P^b^ value	P^c^ value	P^c,d^	P^c,e^	P^c,f^
Age of evaluation (y)	45.4 ± 15.4	45.9 ± 14.8	20.6 ± 5.3	22.6 ± 6.4	0.501	0.120	0.000	–	–	–
BMI (kg/m^2^)	25.1 ± 3.9	23.0 ± 2.1	22.6 ± 3.3	18.6 ± 3.9	0.050	0.004	0.074	–	–	–
HR (beats/min)	73.6 ± 9.9	69.4 ± 10.6	70.0 ± 4.6	72.3 ± 5.6	0.269	0.417	0.404	–	–	–
					Adjusted for age at evaluation and BMI					
IVSd (mm)	7.6 ± 1.1	7.8 ± 1.9	6.4 ± 0.6	7.1 ± 1.6	0.600	0.153	0.009	0.141	0.159	0.173
LVPWd (mm)	7.7 ± 1.0	7.8 ± 1.6	6.4 ± 0.6	6.5 ± 1.4	0.452	0.336	0.005	0.186	0.247	0.237
LVEDD (mm)	46.9 ± 5.1	46.6 ± 3.9	45.1 ± 5.0	42.9 ± 4.5	0.732	0.836	0.398	0.124	0.130	0.141
LVEF (%)	68.4 ± 6.4	70.8 ± 6.1	66.5 ± 7.4	70.4 ± 7.1	0.275	0.314	0.422	0.763	0.939	0.855
LVM (g)	117.9 ± 32.1	120.2 ± 43.9	86.2 ± 20.3	87.5 ± 26.6	0.517	0.288	0.020	0.783	0.888	0.818
LVMI (g/m^2^)	69.2 ± 16.0	71.4 ± 27.0	54.6 ± 6.4	51.3 ± 12.2	0.644	0.824	0.050	0.923	0.943	0.863
RWT	0.33 ± 0.04	0.34 ± 0.07	0.28 ± 0.04	0.31 ± 0.07	0.557	0.369	0.022	0.023	0.032	0.039
E/A ratio	0.9 ± 0.2	NA	1.4 ± 0.1	NA	–	–	0.000	0.043	0.024	0.036

Date represent the means (SD)

^a^: comparison between HP and control A, adjusted for BMI

^b^: comparison between PHP and control B, adjusted for BMI

^c^: comparison between HP and PHP

^d^: *P* value adjusted for gender, age at evaluation, and BMI

^e^: *P* value adjusted for gender, age at evaluation, BMI and serum calcium

^f^: *P* value adjusted for gender, age at evaluation, BMI, and serum phosphorus

Abbreviations: *HP* hypoparathyroidism; *PHP* pseudohypoparathyroidism; *BMI* body mass index; *HR* heart rate; *IVSd* interventricular septum end-diastolic thickness; *LVPWd* left ventricular posterior wall end-diastolic thickness; *LVEDD* left ventricular end-diastolic diameter; *LVEF* left ventricular ejection fraction; *LVM* left ventricular mass; *LVMI* left ventricular mass index; *RWT* relative wall thickness; *E* early diastolic mitral inflow velocity; *A*: late diastolic mitral inflow velocity

### Comparison of echocardiographic parameters between HP and PHP patients

Cardiac parameters reflecting left ventricular structure including IVSd, LVPWd, LVM, and RWT were significantly greater in patients with HP than in PHP, and E/A was lower in the HP group. However, after adjusting for confounders including age at evaluation, gender and BMI, only RWT and E/A showed significant differences (*P* = 0.023 and *P* = 0.042, respectively). Partial correlation analysis showed that serum calcium, serum P and PTH were not correlated with cardiac parameters after adjusting for age at evaluation and gender (Table 2).

### Cardiac conduction in HP and PHP

The resting 12-lead ECG revealed normal sinus rhythm in all patients. The average heart rate was 77.5 ± 12.9 beats/min and 73.3 ± 8.2 beats/min in patients with HP and PHP, respectively. Prolongation of QTc intervals was observed in 8/14 patients diagnosed with HP and in 2/5 patients with PHP, with an average QTc of 473.0 ± 20.3 ms and 455.5 ± 6.4 ms, respectively. In patients with HP and PHP, no significant differences in age of onset, disease course, levels of serum Ca, serum P and serum PTH were detected among those with and without QTc prolongation. PR intervals, QRS duration and ST-T segments were all normal. Holter results revealed normal sinus rhythm in all patients, including 12/15 patients (80%) with supraventricular extrasystoles. Among the 12/15 patients with supraventricular extrasystoles, one (6.7%) female patient with non-surgical HP (aged 64 years with hypertension; disease course 26.2 years; serum Ca, 2.04 mmol/L; and serum P, 1.37 mmol/L) manifested 243 episodes of supraventricular extrasystole in 24 h (the incidence in the remaining 11 subjects ranged from 2 to 57 episodes), as well as supraventricular tachycardia (SVT) (52 episodes, 208 times in total) with complaints of palpitations. Among 7/15 patients (46.7%) with ventricular extrasystoles, one (6.7%) female patient with post-surgical HP (aged 54 years; course 41.2 years; serum Ca, 2.1 mmol/L and serum P, 1.79 mmol/L) presented 4063 episodes of ventricular extrasystoles noticed by 24 h (the remaining six subjects presented with 2–16 episodes) with shortness of breath during activity. No patient showed ventricular tachycardia. No atrioventricular block was found (Table 3).

**Table 3:** Cardiac conduction in patients with HP and PHP

Variables	HP	PHP
12-lead ECG	*N* = 14	*N* = 5
HR (beats/min)	77.5 ± 12.9	73.3 ± 8.2
QTc interval (ms)	449.9 ± 33.9	440.6 ± 16.2
QTc abnormality, n (%)	8/14 (57.1%)	2/5 (40%)
QT (ms)	401.3 ± 33.4	392.5 ± 14.3
QRS interval (ms)	87.5 ± 11.1	92.5 ± 15.4
PR interval (ms)	145.9 ± 18.1	146.5 ± 10.5
24-h Holter ECG	*N* = 10	*N* = 5
24-h total HR (beats)	110,428.0 ± 12,472.5	108,930 ± 11,826.4
Highest HR (beats/min)	122.8 ± 18.3	136.4 ± 12.9
Lowest HR (beats/min)	55.9 ± 3.9	57.2 ± 10.7
Number of supraventricular arrhythmias, n	10/10	2/5
Number of ventricular arrhythmia, n	5/10	2/5

Date represent the means (SD) or N (%)

Abbreviation: *HP* hypoparathyroidism; *PHP* pseudohypoparathyroidism; *ECG* electrocardiograms; *HR* heart rate

## Discussion

In this exploratory study, cardiac morphology, function and conduction were evaluated for the first time in patients with HP (mainly non-surgical HP) and PHP undergoing conventional treatment. The study demonstrated that except for mild QTc prolongation as well as a small percentage of mild arrhythmias, there was no significant difference in left ventricular morphology, systolic dysfunction, or severe cardiac events such as syncope and heart failure in patients with HP and PHP compared with age- and sex-matched healthy controls.

Previous studies suggested that both hypocalcemia and hyperphosphatemia had adverse effects on cardiovascular system [4, 5, 17]. In a recent review of 47 patients, the most common cardiac abnormalities induced by hypocalcemia were heart failure (78%), QTc interval prolongation (38%), and sinus tachycardia (21%) [18]. QTc prolongation has been associated with Torsade de Pointes and sudden cardiac death [19, 20]. In this study, although patients with HP and PHP received regular treatment and maintained low-normal to normal levels of serum calcium, a high proportion of patients (57.1% involving HP and 40% of PHP subjects) showed QTc interval prolongation. Fortunately, arrhythmias were mild in most of our patients, and these patients had no clinical symptoms. Compared with those with severe hypocalcemia from a systemic review and meta-analysis [18], the QTc prolongation in our patients was apparently less severe (449.9 ± 33.9 ms in HP and 440.6 ± 16.2 ms in PHP vs. 510 ms (median) reported in the literature), and the average level of serum calcium in our patients was higher (2.05 ± 0.16 mmol/L in HP and 2.25 ± 0.19 mmol/L in PHP vs. 1.36 mmol/L (median) in published values).

In addition, supraventricular and ventricular extrasystoles were detected in 80% and 46.7% of our subjects evaluated by 24-h Holter electrocardiography. Two patients presented with palpitations or shortness of breath accompanied by supraventricular tachycardia or frequent ventricular extrasystoles. Studies found that ventricular extrasystoles, supraventricular extrasystoles, and supraventricular tachycardia occurred in 40–75%, 60.8%, and 2.2% respectively in healthy subjects via 24- to 48 h Holter examination [21–24]. Even frequent ventricular extrasystoles (more than 60 beats per hour) have been reported in apparently healthy persons [25]. However, evidence suggested that even frequent ventricular extrasystoles did not increase mortality. Kennedy et al. [23] found the risk of death in asymptomatic healthy subjects (*n* = 73, mean age 46.0 ± 13.3 years; 32% with hypertension) with frequent and complex ventricular extrasystoles (63%) for 6.5 ± 1.8 years (from 1 to 9.5 years) was not increased according to US death rates during the same period. In another study, Maurer et al. [26] found that exercise-induced supraventricular tachycardia (6.0% in males and 6.3% in females) in apparently healthy volunteers (843 males and 540 females, aged 20–94 years) did not increase the risk of cardiovascular mortality or coronary events during a follow-up of 5.7 ± 3.9 years compared with control subjects matched for age and sex. However, subjects with SVT appeared to show a higher incidence of spontaneous supraventricular tachyarrhythmia and a higher rate of lone atrial fibrillation than the controls. Due to the small sample size and cross-sectional analysis of this study, it was difficult to assess the severity of arrhythmia in patients with HP and PHP. Thus, further observations and close monitoring are needed to investigate the severity of arrhythmia and related factors in a larger sample size with long-term follow-up.

In this case-control study, no significant differences in left ventricular structure and systolic function were observed between HP and PHP patients with low-normal or normal levels of serum calcium and healthy controls, based on echocardiography. Left ventricular hypertrophy was not observed in all patients, although a few female patients may display borderline concentric remodeling and eccentric hypertrophy, as shown in Fig. 1. An early and small study reported cardiac function in eight HP/PHP patients (aged 13–31 years) with symptomatic hypocalcemia (average serum calcium 1.8 mmol/L; range, 1.33–2.13 mmol/L), and normal M-mode measurements with unknown LVMI and RWT [9]. Acute changes in circulating ionized calcium levels altered myocardial contractility in experimental studies [27]. Clinical studies reported various cardiac events, including acute heart failure and abnormal cardiac structure and function, in HP and PHP patients with severe hypocalcemia (serum calcium range, 0.78–1.55 mmol/L) [28–30]. Hypocalcemia (range, 0.65–1.90 mmol/L) was significantly correlated with LVEF (B = 5.16, *P* <  0.01) in patients with HP [18]. LVEF was normal in patients with hypocalcemia and was not correlated with serum calcium levels probably due to the limited sample size and fewer comorbidities associated with cardiovascular risk. We speculated that the acute and severe hypocalcemia may have different impact on cardiac function due to chronic sub-normal hypocalcemia in relatively well-controlled hypoparathyroidism in this study.

Compared with PHP patients, RWT appeared to be larger in HP patients in our study after adjusting for age at evaluation, BMI, and levels of serum calcium and serum phosphorus. However, they were all within the normal range. The levels of serum calcium, serum phosphorus, and PTH were not related to RWT. In addition, due to the small sample size, patients with post-surgical and non-surgical HP were not separated in our study. Therefore, further classification of large samples of patients diagnosed with HP and PHP into subgroups for comparative analyses may facilitate the determination of differences and clinical significance accurately.

This study has limitations. The major limitation is the small sample size due to the rarity of non-surgical HP and PHP. The cross-sectional design is another limitation, which prevented the analysis of changes in cardiac structure and function during long-term follow-up and precluded any inferences of causal relationship. Also, we have not included patients with previous heart damage due to hypocalcemia, and therefore, the cardiac characteristics of such patients may have been missed. We only included patients who consented to cardiac examination, which suggested a selection bias. Another possible weakness is the relative youth of the included patients since the effects of HP on the cardiovascular system take longer and may be more dramatic in older patients. Meanwhile, date from the controls including biochemical indicators, 12 lead ECG and 24-h Holter electrocardiography were incomplete, although we speculated that the results of the study were not significantly affected. Finally, the patients were not categorized into subgroups according to different etiologies and the small sample size prevented any comparison of differences between subgroups.

## Conclusions

The results of this preliminary study indicate no definitive evidence of a negative effect of cardiac structure or systolic function among patients diagnosed with HP and PHP receiving regular calcium and vitamin D analogs, except for a significant proportion of subjects with prolonged QTc and a few subjects of arrhythmia, warranting further investigation. Studies with a larger sample size are warranted to verify or further explore this situation.

## Data Availability

The datasets generated during and/or analysed during the current study are available from the corresponding author on reasonable request.

## Abbreviations

A: Late diastolic mitral inflow velocity
ASE: American Society of Echocardiography
BMI: Body mass index
BNP: B-type natriuretic peptide
BSA: Body surface area
Ca: Calcium
CAD: Coronary artery disease
CK: Creatine kinase
CKMB: Creatine kinase-myocardial isoenzyme
cTnI: Cardiac troponin I
CVDs: Cardiovascular diseases
E: Early diastolic mitral inflow velocity
ECG: Electrocardiogram
eGFR: Estimated glomerular filtration rate
HP: Hypoparathyroidism
IVSd: Interventricular septum end-diastolic thickness
LVEDD: Left ventricular end-diastolic diameter
LVEF: Left ventricular ejection fraction
LVPWd: Left ventricular posterior wall end-diastolic thickness
P: Phosphorus
PHP: Pseudohypoparathyroidism
PTH: Parathyroid hormone
PUMCH: Peking Union Medical College Hospital
QTc: Corrected QT
RWT: Relative wall thickness
SVT: Supraventricular tachycardia
ULN: Upper limit of normal range
